# 3D imaging and quantitative analysis of adipocytes in situ and ex situ

**DOI:** 10.1080/21623945.2025.2558573

**Published:** 2025-09-21

**Authors:** Isabelle Hue, Adèle Branthonne, Manon Thomas, Violette Thermes, Jérôme Bugeon

**Affiliations:** Laboratoire de Physiologie et Génomique des Poissons, INRAE, Rennes Cedex, France

**Keywords:** Visceral adipocyte, subcutaneous adipocyte, lipid staining, clearing, 3D imaging, 3D image analysis, adipocyte diameter, adipocyte shape

## Abstract

Different adipose tissues (AT) have been described, including subcutaneous and visceral tissues (SCAT and VAT). They display different morphological structures, physiological and metabolic functions. Imaging adipocytes in the whole AT was not feasible because of the large adipocyte sizes and the lipid-full content of the droplets that increased the refractive index. Tissue clearing is then required mainly through a delipidation step, which induces also a tissue shrinkage. Our aim was to image in 3D freshly extracted adipocytes and compare them to those within their tissues. Trout ATs were stained with 5DTAF (extracellular matrix) and Nile Red (lipids). After clearing with Histodenz, 3D images were obtained using a confocal microscope, and adipocytes were segmented and measured. In situ, major differences in adipocyte size and shape were observed between the VAT and SCAT. Ex situ, only the size mattered because all cells were round outside their tissues. This method can be applied to other species, such as mice. In situ, adipocyte sphericity was even higher in the SCAT from a Swiss and a C57Bl6. This approach demonstrates that 3D adipocyte imaging with lipid labeling enables accurate morphological characterization, provides insights into depot-specific structural features, and supports optimization of cell isolation protocols

## GRAPHICAL ABSTRACT

**Figure:**
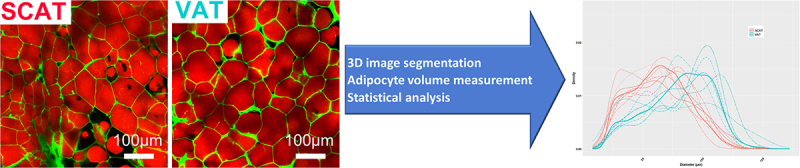

## Introduction

Adipose tissue (AT) is a dynamic tissue of the body that responds to environmental stimuli and adjusts in size accordingly [1]. Therefore, the AT vasculature is essential for nutrient uptake [2]. AT also serves as a growth niche for adipocyte progenitors [3] and harbours adipocyte populations of different sizes, as adipocytes enlarge through lipid uptake (hypertrophy) and storage (triglycerides, mainly TG). Nonetheless, the AT expands by increasing the number of adipocytes (hyperplasia). Both processes occur throughout life, as observed in fish [4]. Depending on their function and cellular specificity, white, beige, and brown adipocytes can be distinguished [5].

The different adipose tissues documented in the literature show slight differences among the species. For instance, subcutaneous and visceral tissues (SCAT and VAT, respectively) have different locations, morphological structures, and physiological and metabolic functions (mice [5,6], and [7]). Subcutaneous tissues harbour more non-adipocyte components than visceral tissues (vessels, collagen fibres, tilapia [8]), whereas the third fat depot (intramuscular adipose tissue, IMAT) contains adipocytes of a smaller size (trout [9]). According to the species, VAT or SCAT appears first (VAT in zebrafish, SCAT in mice [10], and zebrafish [11]). Brown and beige AT, not firmly described in fish, have been ignored so far, even though SCAT from Nile tilapia was recently reported to display a few brown characteristics [8].

Regarding AT morphological structures, histological sections have long been the way to look at those tissues in 2D after haematoxylin-eosin staining or fluorescent labelling Such an approach provides adipocyte numbers and cellular area (µm [2]), but with no lipid content for paraffin sections. There were used for different species using dedicated software [12]: AdipoGauge, mouse data). After adipocyte isolation or culture, fluorescent labelling and flow cytometry have been used to provide lipid droplet or nuclei descriptions, together with cell size or labelling intensity [13]. Labelling probes were LD540, a lipophilic fluorescent dye, to visualize the lipid droplet, Ki-67, a nuclear marker for proliferation, and AdipoQ, an automated image analysis pipeline [14].

Imaging adipocytes in a whole adipose tissue, in 3D, was long unfeasible owing to the large adipocyte sizes and the lipid-full content of the droplets that scattered the fluorescent light and created uneven illumination [15]. As an exception, because of its small size and transparency, the whole zebrafish allows to: i) image multiple adipose tissues after fixation or in real time on living fish [7], and ii) refines the observation of visceral zones [16]. In many other species, tissue-clearing approaches are often required to partially remove the components that scatter or absorb light using a delipidation step [17; 18]. For example, the AdipoClear process adapted from iDISCO/iDISCO+ [19] removes lipids from mouse tissues to visualize nerves, blood cells, and immune cells in 3D [20]. Similarly, Theobalt (2021) used another solvent (3DISCO) to clear and image porcine fat tissues using a light sheet microscope.

In this context, our first aim was to image adipocytes in 3D while preserving their lipid content, both in their original tissue (in situ) and in freshly extracted cells (ex situ), to evaluate the quality of the cell extraction protocol and its suitability for the tissues of interest, SCAT and VAT. The second aim was to use artificial intelligence (AI) segmentation techniques to quantify and compare parameters such as the cell size and shape of in situ and ex situ adipocytes of subcutaneous or visceral origins. Trout tissues were our main focus, but in order to demonstrate that this method could be applied to other species we used the adipose subcutaneous tissue slices from two different mice (breed, age and weight).

## Results

### Fluorescent probe selection for 3D imaging, based on usual 2D imaging of trout AT

Mature adipocytes, with their high lipid content and thin cytoplasmic ring, where the nucleus is often flattened, are challenging to label. However, these cells can be easily identified based on their large lipid droplets. Moreover, VAT and SCAT look different; the first is large, soft, and white, whereas the second is small, thin, and grey, differences that one can still recognize once tissues have been thin-sliced. The easiest option to compare tissues and extracted cells is to label them similarly and visualize the cell membranes, lipids, and nuclei using dual or triple labelling. After testing different co-labelling options on trout tissues and cells (Supp data 1), we finally chose extracellular matrix (ECM)/lipid labelling (5DTAF/Nile Red) for the tissues and cell membrane/lipid labelling (Cellmask/Bodipy) for the extracted cells. These labelling options were first validated in 2D with a fluorescence microscope using tissue sections and cells after fixation. Imaging them in 2D is easy, but extracting their diameters or volumes is not satisfactory because of their spherical shape and broad array of sizes. Imaging sections at the poles of these spheres is confusing because they cannot be sorted between small adipocytes and the poles of large ones, especially within tissues where cells of different sizes are interspersed within the ECM (as illustrated in [21]). Thus, 3D imaging is the only way to achieve this.

### Labelling, clearing, 3D imaging: set-up of the protocol

**Figure 1.**
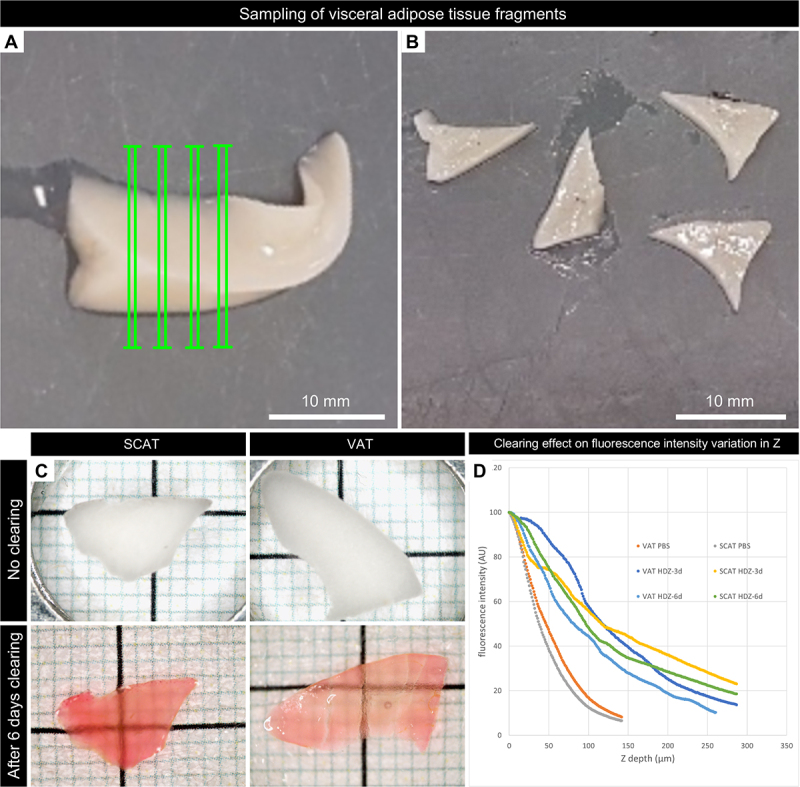
Adipose tissue sampling and clearing. Sampling of visceral adipose tissue of trout. After visceral tissue dissection, (A) thick sections (2 mm) in green were collected, (B) and processed. Clearing of 1324 g trout adipose tissue (SCAT and VAT) with Histodenz: comparison with tissue before clearing (C) and impact on confocal fluorescence intensity (D). Clearing with Histodenz (HDZ) increases imaging depth (Z) within adipose tissues (VAT, SCAT), compared to no clearing at all (PBS). We chose Histodenz clearing for 3 days along the whole study.

After imaging parts of the tissues and cell extractions to select fluorochromes for further use, we came to the objective of this work, namely, the imaging of whole tissue and cells in 3D. To do so, a clearing step was necessary, but the commonly used delipidating agents were not selected here, as our objective was to preserve and visualize lipid droplets. Thus, we opted to use aqueous Histodenz PBS solution. Incubation for approximately 3 days enabled the clearing of both VAT and SCAT (Figure 1(C)), allowing imaging at a depth that was 2–3 times greater along the Z-axis compared to PBS (Figure 1(D)).

### Biological features in situ and ex situ on trout adipocytes; brief extension to two mouse in situ samples

**Figure 2.**
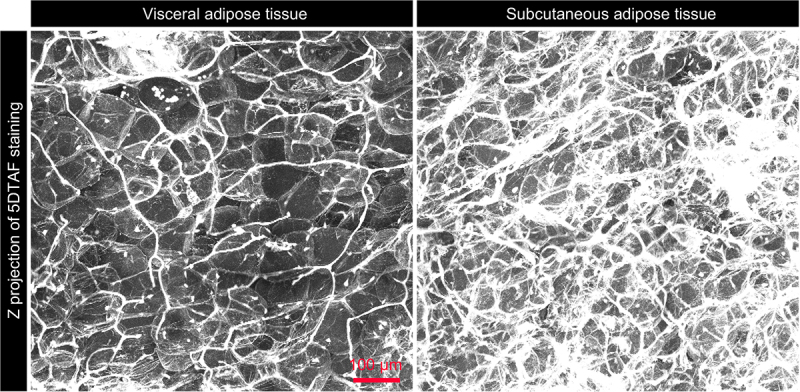
Extracellular matrix staining on cleared VAT and SCAT. A z maximum intensity projection of the VAT and SCAT from a trout of 1304 g labelled with 5DTAF, cleared with Histodenz and observed using a confocal microscope. The 5DTAF staining reveals the extracellular organization (ECM) within both tissues from the same fish. In the VAT, the ECM has a fine, small network, whereas in the SCAT, a denser, thicker network is observed.

**Figure 3.**
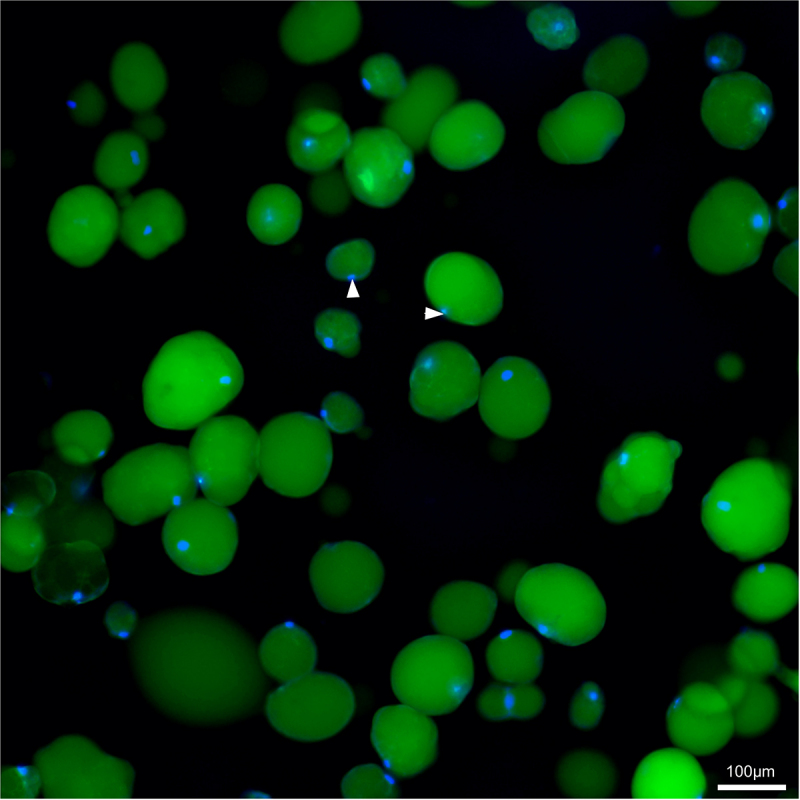
2D view of ex situ SCAT trout adipocytes, stained with Bodipy (green), DAPI (blue); arrows indicate nuclei. Nuclei sign for cells and help distinguish cells from lipid droplets, at the end of the cell extraction. The protocol used is the one we describe here, as previously reported. Bodipy labels the lipids. Adipocytes of different sizes are easily visible (30 to 130 µm for instance). SCAT from a trout of 993 g, out of the M12-M19 experimental design.

**Figure 4.**
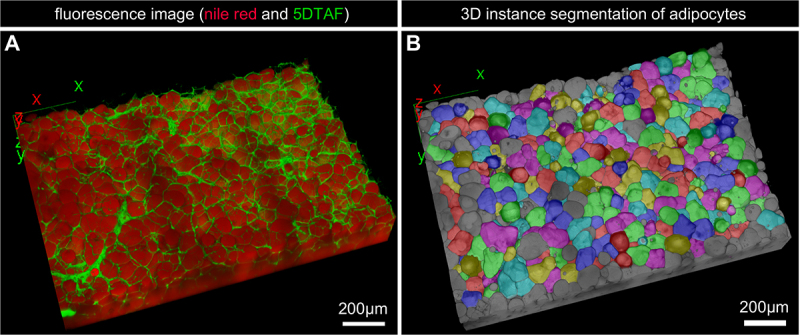
3D view of trout VAT. A) 3D reconstruction of the VAT from a trout of 1250 g labelled with Nile red/5DTAF and observed using a confocal microscope. B) result of the 3D segmentation of individual adipocytes. Only whole adipocytes were measured (in colour), i.e. all adipocytes touching a stack border were discarded (in grey).

Subsequently, 5DTAF staining of cleared VAT and SCAT revealed differences in terms of extracellular matrix (ECM) density and structure (Figure 2). Adipocytes, ‘grey bubbles looking’, were difficult to distinguish under a thick ECM in SCAT. In both tissues a few black holes correspond to missing cells. In whole AT, the nuclei were so numerous that it was impossible to associate any of them with an adipocyte (not shown), thus echoing recent data that reported multiple subpopulations of cells in visceral or subcutaneous ATs (human for instance [22]). In the extracted cells, nuclei were easily imaged in 2D prior to clearing. They attested to cells instead of lipid droplets (Figure 3). Size differences among adipocytes were then clearly visible Complementarily, 3D imaging on thick adipose tissue slices led to individual cell segmentation, such that their volume, shape, and organization within each tissue became measurable and visible (Figure 4).

**Figure 5.**
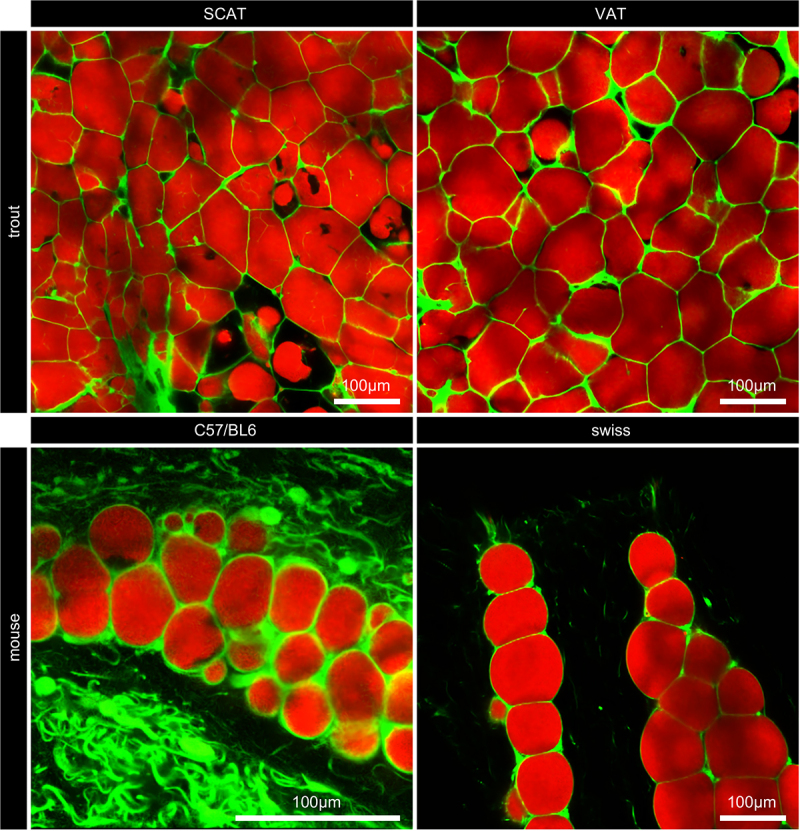
Visceral and subcutaneous AT from trout and mice. Similar labelling (Nile red/5DTAF) and imaging to visualize lipids and extra-cellular matrix organizations from different adipose tissues. Lipids appear all red, uniformly labelled by Nile red. Moreover, extra-cellular matrices look rather different. Defined by these ECM networks (green lines), adipocyte cell shapes look different too: round in mice tissues, angular in trout tissues. Cell sizes also differ much between C57Bl6 SCAT and the other three samples. Trout of 1250 g, C57Bl6: a male mouse of 23 g, Swiss: a female mouse of 48 g.

Easily transferred to mouse adipose tissue slices, our protocol showed a major difference with adipocytes from trout in 2D (Figure 5) and 3D (supp data 4): visually they display a rounder shape. Another difference appeared as well among the two mice samples we looked at (each one originating from a different weight, age and breed) with adipocytes of smaller sizes in the SCAT from the C57Bl6 male (6 weeks old, 23 g) than in that from the Swiss female (32 weeks, 48 g). Used herein to show that our method was usable on other animals than fishes. We have not explored mouse data further, which is only an illustration of our method here Quantitative data extracted from the 3D images of the mouse and trout samples

**Figure 9.**
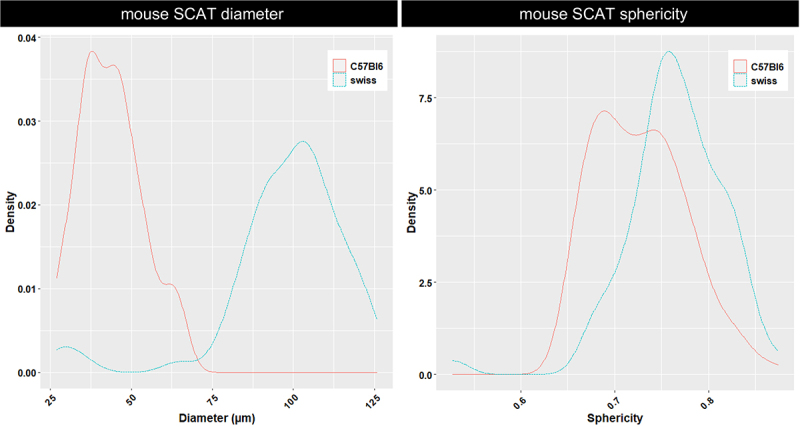
In situ distributions of adipocyte diameter and sphericity in subcutaneous adipose tissue (SCAT) from two mouse strains. Adipocyte diameters, expressed in micrometres (µm), were calculated from 3D adipocytes volumes Sphericity, ranging from 0 to 1 (with 1 corresponding to a perfect sphere), was quantified in 3D to assess cell roundness. Diameter and Sphericity distributions are displayed as probability density functions (density) obtained by kernel density estimation, computed using kernel density estimation with the geom_density() function with ggplot2 R package. Considered here are the data from the C57Bl6 mouse of 23 g and Swiss mouse of 48 g. The cell diameter distribution along the SCAT of the Swiss mouse strictly differs from the one within the SCAT of the other mouse that we used as standard for the trout design (C57Bl6), namely: 90–110 µm vs 38–48 µm). The distributions of their sphericities differ as well (0.77 vs a main peak at 0.68), may be referring to a different ECM network thickness or density as suggested from Figure 6.

Of interest, the subcutaneous adipocytes from the two mice that we used presented different mean diameters (100 µm in the Swiss female, 38 and 50 µm in the C57Bl6 male) together with a close sphericity: 0.78 (Swiss) and 0.73 for one of the two adipocyte peaks from C57BL6 (Figure 9). However, a smaller sphericity was observed for the other peak of C57Bl6 adipocytes (0.68), as shown in Figure 5, through the intra-tissular trapezoidal shapes of most of these adipocytes.

Because of the ECM context within the tissues and the aqueous context for the extracted cells, we further explored the relationship between cell size and cell shape in situ among trout fat types. Size and sphericity ranges were equivalent (Supp data 2) but sample distributions differed: compact for 70–80% of VAT samples (60–120 µm; 0.5–0.8), broader for SCAT (20–120 µm; 0.4–0.8).

Based on the experimental design for the trout samples, the last step was to extract quantitative data from the in situ and ex situ samples, namely: the subcutaneous and visceral cell types from the trout batches of 0.75 or 1.1 kg. Altogether, 24 tissues and 20 extracts of approximately 10,000 cells each were analysed (details in the Materials and Methods section).

**Figure 6.**
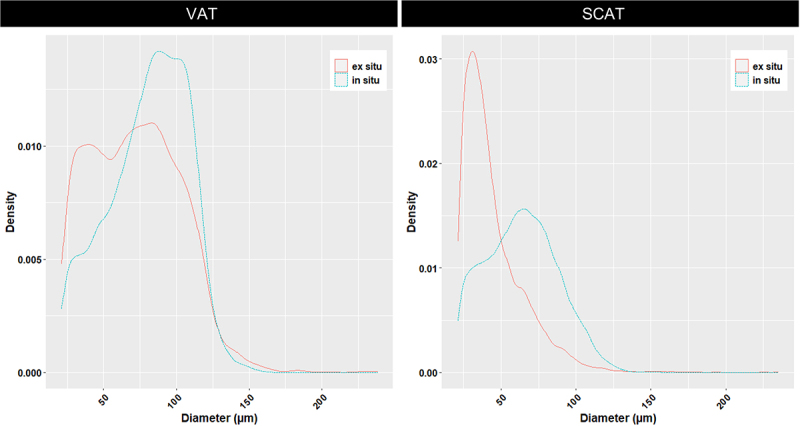
Distributions of in situ/ex situ diameters for VAT and SCAT.

**Figure 7.**
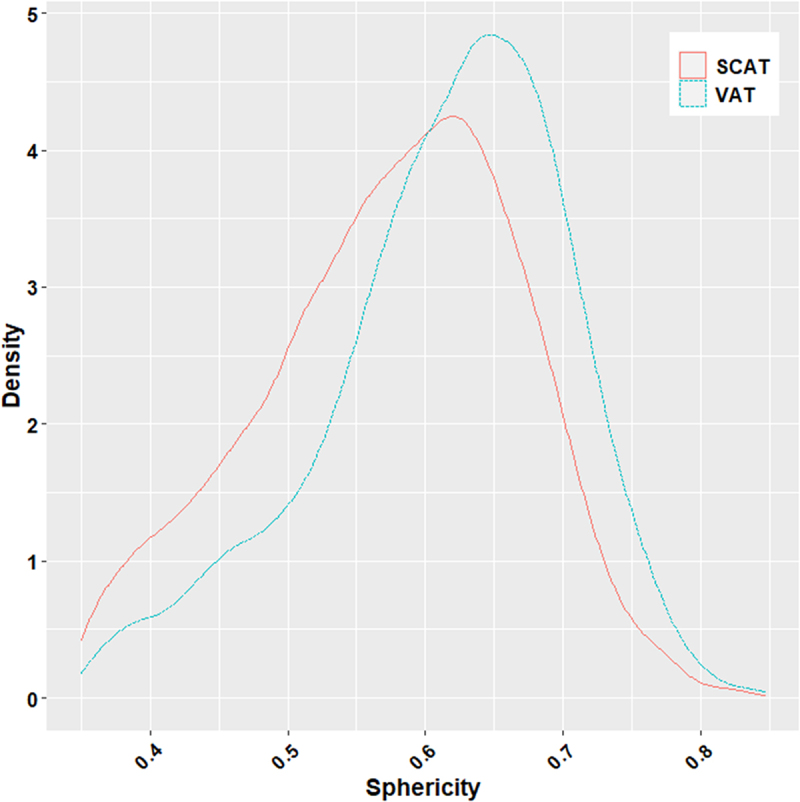
In vivo adipocyte sphericity according to VAT and SCAT. Adipocyte sphericity, ranging from 0 to 1 (with 1 corresponding to a perfect sphere), was quantified in 3D to assess cell roundness. Sphericity distributions are displayed as probability density functions (density) obtained by kernel density estimation, computed using kernel density estimation with the geom_density() function with ggplot2 R package. Considered here are the whole in situ datasets (details in suppdata 1). in situ, the trout visceral adipocytes display altogether a higher mean sphericity than the subcutaneous ones (0.64 vs 0.62).

**Figure 8.**
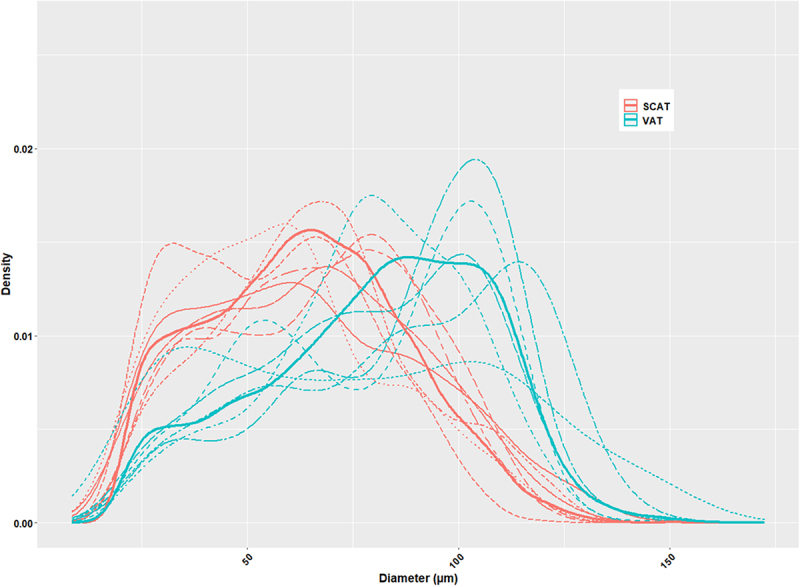
Diameter distribution of in vivo adipocytes according to fishes and tissues. Adipocyte diameters, expressed in micrometres (µm), were calculated from 3D adipocytes volumes. Data were obtained from both VAT and SCAT in 12 individual trout. Diameter distributions are represented as probability density functions estimated by kernel density estimation (geom_density() function, ggplot2 package in R), with dotted curves representing individual fish and solid lines indicating the mean distribution per tissue type. A two-sample Kolmogorov – Smirnov test revealed a highly significant difference between tissues (*p* < 0.001), with mean diameters of 63.88 µm for SCAT and 81.32 µm for VAT (*p* < 0.001).

**In situ**, globally looking at the mean diameter according to fat-types, visceral adipocytes were larger than subcutaneous ones (81.32 vs 63.88 µm; Figure 6), with a slightly higher sphericity (0.64 vs 0.62; Figure 7). On an experimental basis, the distributions of adipocyte diameters differed among tissues, with the largest adipocytes in VAT (150–200 µm), highest densities for a large diameter (120 µm) in VAT, and for a small diameter (30 µm) in SCAT, together with a reduced variability within the density parameter for the SCATs (Figure 8).

Adipocyte diameters, expressed in micrometres (µm), were inferred from 3D measured cell volumes. The datasets include both in situ and ex situ conditions (*n* = 10), each comprising a sufficient number of adipocytes to allow reliable estimation of mean distributions. Diameter distributions are represented as probability density functions (density), computed using kernel density estimation with the geom_density() function with ggplot2 R package. A two-sample Kolmogorov – Smirnov test revealed a highly significant difference between in situ and ex situ diameter distribution for both tissues (*p* < 0.001)

In situ, adipocytes from VAT appear larger than ex situ (40–150 µm vs 25–125 µm, left panel). In situ, adipocytes from SCAT appear larger than ex situ. (25–150 µm vs 25–100 µm, right panel)

**Ex situ**, we globally observed over the experiments where we considered that tissue extractions resulted in adipocytes with a smaller mean cell diameter and a higher cell sphericity, thus evidencing a stronger difference among fat-types in situ than ex situ. For instance, SCAT extractions (ex situ) contained three times fewer adipocytes (mean diameter < 60 µm; density: 0.030; Figure 6) than VAT extractions, and five times fewer than larger ones ( > 80 µm, density: 0.002; Figure 6). On a per tissue basis, VAT-extracted cells were indeed closer to in situ features (as evaluated through mean diameter and cell diameter distribution), with only one-fourth of the 70–110 µm population missing. Simultaneously, an increase in the 20–60 µm population appeared by doubling it (Figure 6). To improve SCAT adipocyte recovery during the extraction process we tried a filtering on 300 µm, instead of 200 µm, and processed the cells as those from VAT. Unfortunately, this did not allow the recovery of larger cells, and the diameters of the recovered adipocytes were similar.

## Discussion

Our imaging procedure conserved lipids within the cells of visceral and subcutaneous fat tissues in contrast to the use of solvents such as iDISCO/iDISCO+ [19] or 3DISCO [18] to clear and image fat tissues. Relying on an upright confocal microscope and Cellpose as a deep learning segmentation algorithm, we demonstrated for the first time the 3D size and shape of adipocytes in rainbow trout and mouse. Moreover, no tissue shrinkage occurred compared to the data, for which a volume shrinkage of approximately 21% occurred because of the clearing process [18]. For in situ samples, imaging of a large number of adipocytes along the Z-axis was not possible because of the limited clearing capacity of the Histodenz. Therefore, it was necessary to acquire XY mosaics to capture a sufficient number of adipocytes, especially larger ones. By adjusting the fluorescent probes instead of antibodies, our procedure was easily transferable from tissues to ex situ extracted cells prior to any culture (extracellular matrix and lipids: 5DTAF, Nile Red *versus* cell membranes and lipids: cell mask, Bodipy). Easily transferable too from trout tissues to mouse SCAT (Figure 5).

The four fluorescent probes, selected on a larger basis (Supplementary Data 1), mainly relied on previous studies. First, the 5DTAF probe was used because ECM labelling was reported to be easier to use than membrane labelling in zebrafish adipose tissues [23]. Neutral lipids (such as triacylglycerol & sterols esters) marking late adipocyte differentiation, Nile red was often preferred to Lipidtox as it labels the wide range of LDs sizes from very small to very large [11,24]. Reflecting a biological process in which LD size reflects the balance between lipogenesis and lipolysis, that is, lipid incorporation into LDs versus lipid mobilization from LDs, this wide range of cell sizes is of interest, and each process is complex and regulated at multiple levels [10,25]. With the ECM digested during cell extraction, the Cell Mask was ideal for imaging and analysing ex situ extracted adipocytes and sorting out the cells from free lipids (lipid droplet alone). Bodipy completed the panel for whole lipid labelling [24; 26], as opposed to Lipidtox, which helps to visualize different lipid contents [9,11]. As for the nuclei, Hoechst or Dapi labelling [23; 25,27] followed by imaging confirmed the cellular status of what could appear as labelled fluorescent beads. Two-dimensional imaging, which we used backwards for biological reasons such as nuclei checking prior to clearing, was for a long time the only method to look at adipocytes in situ and ex situ. Adipocyte numbers and cellular areas, as observed in tissue sections, were provided by dedicated software, such as AdipoGauge [12], AdipoQ [14], and AdipoSoft [28]. Confocal or light-sheet microscopy, together with tissue clearing and 3D mounting, allows 3D imaging and unbiased adipocyte volume quantification.

As a result, these adipose tissues appeared different with i) a dense ECM that likely reduced cellular sphericity owing to higher physical constraints in the subcutaneous tissue compared to (ii) a larger adipocyte diameter in the visceral tissue. Indeed, the passive mechanical properties of murine VAT and SCAT differ [29]. Moreover, VAT was often reported as morphologically different from SCAT, with a larger mean diameter in rainbow trout [30], as well as in mouse [31]. Conversely, SCAT contains adipocytes heterogeneous in size, which could appear as two main populations (20–50 µm vs 100–200 µm [28,32]); or as two distinct layers: a superficial layer formed of small fat lobules and a deeper layer formed by larger lobules, rather lipolytic, as in humans [33; 34]. Concomitantly, cells extracted from these tissues (‘ex situ adipocytes’) also differed, with visceral adipocytes displaying a larger diameter than subcutaneous adipocytes. As in most papers [35–37], ex situ refers herein to ‘ex vivo prior to any seeding’ as opposed to the ex situ that relates to different times in culture [38]. The ‘less than 25 µm excluded-cells’ from [38] correspond here to only a small subset of the adipocyte population, which exhibits a mean diameter of 40 µm. Nonetheless, the in situ *vs* ex situ distributions were closer for mean visceral diameters than for subcutaneous diameters. Two hypotheses can be proposed: i) the dense ECM of the SCAT makes adipocytes fragile after extraction, or ii) collagenase digestion, which initiates the extraction phase, is a critical step, during which adipocytes may rupture and coalesce into fat droplets (human [36]; mouse [39]). However, such a scenario has not yet been proposed. However, in the few studies that isolate different adipocyte types within the same study, extraction protocols sometimes differ in their use of collagenase [40] for instance), a parameter we could easily test later on to refine our protocols. Independent of cell extraction *per se*, visualizing differences in ECM/adipocyte ratios among trout adipose tissues is of utmost interest for future approaches, such as fat explants or 3D cell cultures, which have already been initiated in mammals [15,41,42].

Altogether, the experimental design that we used on 24 tissues and 20 of the 24 corresponding cell extractions allowed us to identify, at first, a significant difference between adipose tissues, which we started exploring herein, working with large sampling of tissues and extracted cells, namely: 15462 vs 10,484. Another difference between fish weights appeared as well, less significant in the current design (0.75 kg vs 1.1 kg) than along the continuous trout growth reported by Rosell-Moll (2025), who compared adipocyte features from trout of 0.3 kg and 2 kg. Our data revealed that the in situ distribution of adipocyte diameters differed markedly between tissues and fish (Figure 8). Nonetheless, variability among fishes appeared lower for SCATs than for VATs, which may be related to tissue-dependent differences in adipocyte hyperplasia/trophy, ECM stiffness, or broader physiological differences across individuals. Last but not least, easily transferable to mouse adipose tissues, confirming previous adipose cell sizes reported for males of about 8 weeks (C57Bl6 [43; 44]) or females of 32 weeks (Swiss [45]), our protocol opens new avenues for interspecies comparisons., and likely : in terms of anatomy, metabolism, gene expression profiles, and ex vivo culturing features [31; 46].

## Methods

### Animals

Male and female rainbow trout (*Oncorhynchus mykiss*) were reared in a circulating rearing system at the experimental facilities of the Fish Physiology and Genomics Laboratory (http://doi.org/10.15454/45d2-bn67) under a natural simulated photoperiod, at 12°C±1°C (pH 7.8–8.4; NH4 < 0.1 mg/L). Fish were fed ad libitum a commercial diet (Le Gouessant) and raised in tanks of 300 or 1000 L (0.75 or 1.10 kg batches, respectively). For tissue collection, fish anesthetized with tricaine (50 mg/ml) and euthanized with tricaine (200 mg/ml) were weighed. The facility (LPGP, Rennes, France) has permit number D35–238–6, delivered by French veterinary services. Fishes used for preliminary experiments and methodological settings, helped preparing the experimental design described below. Mice (*Mus musculus*) from the Swiss and the C57Bl6 lines, under standard housing (22°C ± 2°C, open cages of 5, sawdust litter, water at will) and feeding conditions (standard diet at will, 2918 INOVIT), were kindly provided to us by colleagues from the ARCHE Animal Facility (Rennes 1 University, France). From them, we used a few and present herein images from a C57Bl6 male of 6 weeks (23 g) and a Swiss female of 32 weeks (48 g). Under regular diet, similar weights were reported for C57Bl6 mice [47; 48] or Swiss mice [49] that did not class those animals as obese or lean but ‘within the dedicated JANVIER’s growth curve’

### Adipose tissue fixation, labelling, clearing

Visceral and dorsal subcutaneous adipose tissues (VAT; SCAT) from a few trouts used for preliminary tests or from the two trout batches (±750 g; ±1100 g) of the experimental design were dissected, and for a small part, cut into regular slices using a homebuilt tool made with two parallelized razor blades (Figure 1(A, B)). The slices were fixed overnight in PFA 4% at 4°C, washed in PBS 1x and stored in PBS-Azide 0.05%PFA 0.5% at 4°C. Rinsed in PBS 1x prior to labelling with 5-DTAF (200 µg/ml, 8 h) in 0.1 M sodium bicarbonate pH 9 [50], they were rinsed again with PBS 1X prior to Nile Red labelling (1/500, overnight). Samples were then cleared in 50% Histodenz/50% PBS for 3 h under agitation, prior to additional baths of Histodenz 100% (*n* = 3, over 2.5 days, Refracting Index: 1.4571). The subcutaneous adipose tissues come from the 2 mice that we used (a C57Bl6 male of 23 g, 6 weeks and a Swiss Female of 48 g, 32 weeks) were processed in the very same way. The product references have been compiled in Supplementary Data 1.

### Adipocyte extraction, labelling, clearing

From the pieces of tissue left over by tissue slicing, mature adipocytes were extracted from VAT, as published by [51] and from SCAT, as reported in [38]. Briefly, adipose tissues were cut into pieces and incubated with collagenase II (125 U/ml; Sigma C6885) in KBS 1x (Krebs-Hepes-BSA1%) on a shaking platform at 17°C for 90 min. Visceral and subcutaneous cellular suspensions were filtered at 300 and 200 µm, respectively, prior to successive washes by flotation in KBS 1X-BSA1% (*n* = 2) and KBS 1X-BSA2% (*n* = 2). The cells were rinsed twice in PBS 1x after permeabilizing (Saponin: 1 mg/ml, 10 min, room temperature) and prior to co-labelling with Cell Mask deep red and Bodipy (1/800 each, 2h30), adipocytes were then rinsed again and loaded into agarose wells formed by a Stampwell mould from Idylle-labs (7 µl/slot) using a low gelling agarose at 1.5%. They were then cleared (3 slots) in Histodenz 50% for 3 h and additional baths of Histodenz 100% (*n* = 3, over 2.5 days, refraction index: 1.4571).

### Confocal imaging

3D imaging was performed using a Leica TCS SP8 laser scanning confocal microscope, equipped with a HC Fluotar L 25x/1.00 IMM (ne = 1.457) MotCORR VISIR objective for cleared samples and a HC FLUOTAR L 25x/0,95 W VISIR objective for PBS acquisitions. Cleared tissues and cleared cells mounted on low-gelling agarose slices were immobilized at the bottom of a 60-mm Petri dish with a tiny drop of superglue. The dish was filled with Histodenz and covered with mineral oil to avoid evaporation, and thus, refractive index changes during acquisitions. For optimal refractive-index homogenization, fixed samples were mounted the day before imaging. Images were acquired with the pinhole set to 1 Airy unit (55.8 µm). Sequential scanning mode was applied with photomultiplier detectors (PMT). 5DTAF was excited with a 488 nm laser (1% intensity) and emission collected between 493 and 523 nm (gain 725). Nile Red was excited with a 552 nm laser (0.6% intensity) and emission was collected between 558 and630 nm (gain 700). The voxel size was 1.15x1.15x1.15 µm. Tissue imaging was performed over a depth of 300 µm using a 2 × 3 imaging mosaics.

### Image analysis

To obtain individual adipocyte measures, such as volume or 3D shape, image stacks were analysed using Cellpose 2 to specifically segment the adipocytes. As described in [52], a human-in-the-loop process was used to train a new model adapted to segmented adipocyte cells stained and imaged using our protocol (Supplementary Data 4).

A set of eight images was used, and the segmentation obtained with the cyto2 model was manually corrected using GUI, as proposed by Cellpose2. A total of 1694 adipocyte masks were obtained, and the cyto2 model was retrained on manually annotated images (Supplementary Data 3 as an example; 200 epochs, learning rate 0.1, weight decay 0.0001). The model was set with the green channel (5DTAF) as chan and the red channel (Nile red) as chan2, and a diameter of 60 µm was used for inference in 3D. For training and inference, a Dell Precision 7920 PC Intel Xeon Silver CPU with 512 Go RAM and RTX A6000 GPU was used.

As the images of the mask of the adipocyte contained some false positives and not entirely segmented adipocytes on the stack border, a homemade Fiji macro was used with the MorpholibJ plugin to filter the adipocytes and eliminate false positives or not well-segmented adipocytes. First, the masks touching the stack border were discarded, and masks presenting a sphericity under 0.35 and a volume under 5000 voxels were eliminated. Finally, each stack was manually checked in 2D and 3D using a 3D viewer to validate segmentation quality.

The validated images were measured, and the individual volume of each adipocyte was obtained. An equivalent adipocyte diameter was calculated under the assumption that the shape corresponded to a sphere, using the formula $\mathrm{Diameter}=2\sqrt[{3}]{\left(3\times \mathrm{Volume}/4\times \pi \right)}$. The sphericity of each adipocyte was measured using the formula: $\mathrm{sphericity}=\frac{36\Pi v\mathrm{olum}e^{2}}{\mathrm{surfac}e^{3}}$The Individual diameter and circularity were used to compare the adipocyte size distribution between the tissues. For ex situ adipocytes, a Cyto2 model without retraining was used, with only the green channel and a diameter of 40.

### Statistical analyses

All analyses were performed using the RStudio Statistical Software (V 2024.09.1 Build 394). A linear model was used to analyse the effects of tissue, sex, and body weight on the mean adipocyte diameter and shape. The individual and averaged distributions of adipocyte diameters were estimated using kernel density estimation as implemented in the *geom_density()* function of the *ggplot2* package in R. Smoothed probability density functions for each sample as well as their mean were obtained in order to compare adipocyte diameter and sphericity distributions across experimental conditions. To compare distributions of trout adipocyte diameters between the two tissues (SCAT and VISC) an inferential statistic test was performed using a permutation-based two-sample Kolmogorov – Smirnov test (KS). The observed KS statistic was compared with the distribution obtained from 10,000 permutations. The p-value was calculated using the Phipson and Smyth correction to avoid zero estimates.

### Experimental design

The trouts used herein originated from two batches of 0.75 and 1.1 kg composed of four males and four females each. Visceral and subcutaneous adipose tissues were quickly dissected, weighed, and cut into regular slices prior to tissue fixation or pooled for cell extraction and counting. Interestingly, under bright field (ix70, Olympus), without any labelling, the cells extracted from VATs appeared less concentrated (±2.5 x10^6^ cells/g of tissue) but larger (±70–80 µm), when those from SCATs were rather dense and small (±6 x10^6^ cells/g of tissue; ±15–20 µm). Similar to the tissue slices, we fixed, rinsed, and labelled the extracted cells, evaluated several fluorescent molecules in the tissues, and extracted cells after clearing.

Considering male and female trout from two batches of different mean weights, two fat tissues per fish, and six independent experiments, we gathered 24 tissues, all used, and 24 cell extractions, 20 of which were usable only (Supp data 5). In the other 4 subcutaneous adipocytes extracted from M12-919 g, M13-874 g and both M19 fishes (1052 g; 665 g), too few cells have been imaged and analysed (*n* < 100) so that considering them was negligible. Nonetheless, in situ and ex situ analyses were performed on 3D: 15462 and 10,484 adipocytes, respectively. Therefore, the two main factors that affected the mean adipocyte diameter were adipose tissue type (*** *p* < 0.001) and fish weight (* *p* < 0.01). In the present study, the sex of the fish was not significant.

## Supplementary Material

Suppdata1.docx

Suppdata4.docx

Suppdata5.docx

suppdata3.tif

suppdata2.tif

## Data Availability

The datasets produced were uploaded to the Bioimage Archive, and the results of the analysis in Data INRAE:

## References

[1] Hausman DB, DiGirolamo M, Bartness TJ, et al. The biology of white adipocyte proliferation. Obes Rev [Internet]. 2001 [cited 2025 Feb 7];2(4):239–14. doi: 10.1046/j.1467-789X.2001.00042.x 12119995

[2] Blaszkiewicz M, Willows JW, Johnson CP, et al. The importance of peripheral nerves in adipose tissue for the regulation of energy balance. Biol [Internet]. 2019 [cited 2025 Jan 31];8(1):10. doi: 10.3390/biology8010010 PMC6466238 30759876

[3] Ioannidou A, Fisher RM, Hagberg CE. The multifaceted roles of the adipose tissue vasculature. Obes Rev [Internet]. 2022 [cited 2025 Jan 31];23(4):e13403. doi: 10.1111/obr.13403 34866318

[4] Fauconneau B, Andre, S., Chmaitilly, J., Le Bail, P. Y, Krieg, F., Kaushik, S.J. Control of skeletal muscle fibres and adipose cells size in the flesh of rainbow trout.

[5] Hou B, Zhao Y, He P, et al. Targeted lipidomics and transcriptomics profiling reveal the heterogeneity of visceral and subcutaneous white adipose tissue. Life Sci [Internet]. 2020 [cited 2025 Jan 31];245:117352. Available from: https://linkinghub.elsevier.com/retrieve/pii/S0024320520300990 32006527 10.1016/j.lfs.2020.117352 PMC7988272

[6] Pellegrinelli V, Carobbio S, Vidal-Puig A. Adipose tissue plasticity: how fat depots respond differently to pathophysiological cues. Diabetologia [Internet]. 2016 [cited 2025 Jan 31];59(6):1075–1088. doi: 10.1007/s00125-016-3933-4 27039901 PMC4861754

[7] James E.N. Minchin, John F. Rawls. Chapter 3 - In vivo Analysis of White Adipose Tissue in Zebrafish. In: H. William, Detrich, Monte, Westerfield, Leonard I., Zon editors. Methods in cell biology. Elsevier; 2011 [cited 2025 Jan 31]. p. 63–86. Available from: https://linkinghub.elsevier.com/retrieve/pii/B9780123813206000035 10.1016/B978-0-12-381320-6.00003-5 PMC4846293 21951526

[8] Wang Y-W, Zhang J-L, Jiao J-G, et al. Physiological and metabolic differences between visceral and subcutaneous adipose tissues in Nile tilapia *(Oreochromis niloticus)*. Am J Physiol-Regul, Intgr Comp Physiol [internet]. 2017 [cited 2025 Jan 31];313(5):R608–19. doi: 10.1152/ajpregu.00071.2017 28814390

[9] Rosell-Moll E, Ntk M, Balbuena-Pecino S, et al. Morphofunctional characterization of the three main adipose tissue depots in rainbow trout (Oncorhynchus mykiss). Comp Biochem Physiol Part B. 2025 [cited 2025 Jan 31];275:111039. Available from: https://linkinghub.elsevier.com/retrieve/pii/S1096495924001064 10.1016/j.cbpb.2024.111039 39396638

[10] Tandon P, Wafer R, Minchin JEN, et al. Adipose morphology and metabolic disease. J Exp Biol [Internet] 2018 [cited 2025 Jan 31];221(Suppl_1):jeb164970. Available from: https://journals.biologists.com/jeb/article/221/Suppl_1/jeb164970/33998/Adipose-morphology-and-metabolic-disease 29514883 10.1242/jeb.164970

[11] Lepanto P, Levin-Ferreyra F, Koziol U, et al. Insights into *in vivo* adipocyte differentiation through cell-specific labeling in zebrafish. Biol Open [Internet]. 2021 [cited 2025 Jan 31];10(9):bio058734. Available from: https://journals.biologists.com/bio/article/10/9/bio058734/272134/Insights-into-in-vivo-adipocyte-differentiation 34409430 10.1242/bio.058734 PMC8443861

[12] Yosofvand M, Liyanage S, Kalupahana NS, et al. AdipoGauge software for analysis of biological microscopic images. Adipocyte [Internet]. 2020 [cited 2025 Jan 31];9(1):360–373. doi: 10.1080/21623945.2020.1787583 32654628 PMC7469447

[13] Stenkula KG, Erlanson-Albertsson C. Adipose cell size: importance in health and disease. Am J Physiol-Regul, Intgr Comp Physiol [Internet] 2018 [cited 2025 Jan 31];315(2):R284–95. doi: 10.1152/ajpregu.00257.2017 29641234

[14] Sieckmann K, Winnerling N, Huebecker M, et al. AdipoQ—a simple, open-source software to quantify adipocyte morphology and function in tissues and in vitro. MBoC [Internet]. 2022 [cited 2025 Jan 31];33(12):br22. doi: 10.1091/mbc.E21-11-0592 35947507 PMC9635306

[15] Varlamov O, Somwar R, Cornea A, et al. Single-cell analysis of insulin-regulated fatty acid uptake in adipocytes. Am J Physiol-Endocrinol Metab [internet]. 2010 [cited 2025 Jan 31];299(3):E486–96. doi: 10.1152/ajpendo.00330.2010 20570821 PMC2944284

[16] Minchin JEN, Rawls JF. A classification system for zebrafish adipose tissues. Disease Model Mechanisms [Internet] 2017 [cited 2025 Jan 31]:dmm.025759. Available from: https://journals.biologists.com/dmm/article/doi/10.1242/dmm.025759/257211/A-classification-system-for-zebrafish-adipose 10.1242/dmm.025759 PMC5482999 28348140

[17] Vieites-Prado A, Renier N. Tissue clearing and 3d imaging in developmental biology. Devel [Internet]. 2021 [cited 2025 Jan 31];148(18):dev199369. Available from: https://journals.biologists.com/dev/article/148/18/dev199369/272368/Tissue-clearing-and-3D-imaging-in-developmental 10.1242/dev.199369 PMC8497915 34596666

[18] Theobalt N, Hofmann I, Fiedler S, et al. Unbiased analysis of obesity related, fat depot specific changes of adipocyte volumes and numbers using light sheet fluorescence microscopy. PLOS ONE [Internet]. 2021 [cited 2025 Feb 7];16(3):e0248594. doi: 10.1371/journal.pone.0248594 33725017 PMC7963095

[19] Chi J, Crane A, Wu Z, et al. Adipo-clear: a tissue clearing method for three-dimensional imaging of adipose tissue. JoVe [Internet] 2018 [cited 2025 Jan 31];(137):58271. Available from: https://app.jove.com/v/58271 30102289 10.3791/58271 PMC6126572

[20] Geng J, Zhang X, Prabhu S, et al. 3D microscopy and deep learning reveal the heterogeneity of crown-like structure microenvironments in intact adipose tissue. Sci Adv. 2021 [cited 2025 Aug 25];7(8):eabe2480. doi: 10.1126/sciadv.abe2480 33597245 PMC7888944

[21] Lenz M, Roumans NJ, Vink RG, et al. Estimating real cell size distribution from cross-section microscopy imaging. Bioinf [Internet]. 2016 [cited 2025 Aug 25];32(17):i396–404. Available from: https://academic.oup.com/bioinformatics/article/32/17/i396/2450761 10.1093/bioinformatics/btw431 27587655

[22] Maniyadath B, Zhang Q, Gupta RK, et al. Adipose tissue at single-cell resolution. Cell Metab [Internet]. 2023 [cited 2025 Feb 7];35(3):386–413. Available from: https://linkinghub.elsevier.com/retrieve/pii/S1550413123000384 36889280 10.1016/j.cmet.2023.02.002 PMC10027403

[23] Minchin JEN, Dahlman I, Harvey CJ, et al. Plexin D1 determines body fat distribution by regulating the type V collagen microenvironment in visceral adipose tissue. Proc Natl Acad Sci USA [Internet] 2015 [cited 2025 Feb 7];112(14):4363–4368. doi: 10.1073/pnas.1416412112 25831505 PMC4394244

[24] Ott RK, Williams IH, Armstrong AR. Improved whole-mount immunofluorescence protocol for consistent and robust labeling of adult *Drosophila melanogaster* adipose tissue. Biol Open [Internet]. 2024 [cited 2025 Jan 31];13(8):bio060491. Available from: https://journals.biologists.com/bio/article/13/8/bio060491/361348/Improved-whole-mount-immunofluorescence-protocol 39041865 10.1242/bio.060491 PMC11317099

[25] Berger E, Géloën A. Insulin prevents fatty acid induced increase of adipocyte size. Adipocyte [Internet]. 2022 [cited 2025 Jan 31];11(1):510–528. doi: 10.1080/21623945.2022.2107784 35946137 PMC9450899

[26] Laber S, Strobel S, Mercader JM, et al. Discovering cellular programs of intrinsic and extrinsic drivers of metabolic traits using LipocyteProfiler. Cell Genomics [internet]. 2023 [cited 2025 Jan 31];3(7):100346. Available from: https://linkinghub.elsevier.com/retrieve/pii/S2666979X23001210 37492099 10.1016/j.xgen.2023.100346 PMC10363917

[27] Tchoukalova YD, Harteneck DA, Karwoski RA, et al. A quick, reliable, and automated method for fat cell sizing. J Lipid Res [Internet]. 2003 [cited 2025 Jan 31];44(9):1795–1801. Available from: https://linkinghub.elsevier.com/retrieve/pii/S0022227520337457 12777477 10.1194/jlr.D300001-JLR200

[28] Galarraga M, Campión J, Muñoz-Barrutia A, et al. Adiposoft: automated software for the analysis of white adipose tissue cellularity in histological sections. J Lipid Res [Internet]. 2012 [cited 2025 Jan 31];53(12):2791–2796. Available from: https://linkinghub.elsevier.com/retrieve/pii/S0022227520418139 22993232 10.1194/jlr.D023788 PMC3494244

[29] Cesanelli L, Minderis P, Degens H, et al. Passive mechanical properties of adipose tissue and skeletal muscle from C57BL/6J mice. J Mech Behav Biomed Mater [Internet]. 2024 [cited 2025 Jan 31];155:106576. Available from: https://linkinghub.elsevier.com/retrieve/pii/S175161612400208X 38744119 10.1016/j.jmbbm.2024.106576

[30] Weil C, Sabin N, Bugeon J, et al. Differentially expressed proteins in rainbow trout adipocytes isolated from visceral and subcutaneous tissues. Comp Biochem And Physiol Part D [Internet]. 2009 [cited 2025 Jan 31];4(3):235–241. Available from: https://linkinghub.elsevier.com/retrieve/pii/S1744117X09000458 10.1016/j.cbd.2009.05.002 20403757

[31] Börgeson E, Boucher J, Hagberg CE. Of mice and men: pinpointing species differences in adipose tissue biology. Front Cell Dev Biol. 2022 [cited 2025 Jan 31];10:1003118. doi: 10.3389/fcell.2022.1003118/full 36187476 PMC9521710

[32] McLaughlin T, Sherman A, Tsao P, et al. Enhanced proportion of small adipose cells in insulin-resistant vs insulin-sensitive obese individuals implicates impaired adipogenesis. Diabetologia [Internet]. 2007 [cited 2025 Feb 7];50(8):1707–1715. doi: 10.1007/s00125-007-0708-y 17549449

[33] Baptista LS, Silva KR, Jobeili L, et al. Unraveling white adipose tissue heterogeneity and obesity by adipose stem/stromal cell biology and 3D culture models. Cells [Internet]. 2023 [cited 2025 Jan 31];12(12):1583. doi: 10.3390/cells12121583 37371053 PMC10296800

[34] Smith U. Effect of cell size on lipid synthesis by human adipose tissue in vitro. J Lipid Res [Internet]. 1971 [cited 2025 Jan 31];12(1):65–70. Available from: https://linkinghub.elsevier.com/retrieve/pii/S002222752039547X 4322518

[35] Björnheden T, Jakubowicz B, Levin M, et al. Computerized determination of adipocyte size. Obes Res [Internet]. 2004 [cited 2025 Jan 31];12(1):95–105. doi: 10.1038/oby.2004.13 14742847

[36] Guo W, Bigornia S, Leizerman I, et al., et al. New scanning electron microscopic method for determination of adipocyte size in humans and mice. Obes [Internet]. 2007 [cited 2025 Jan 31];15(7):1657–1665. doi: 10.1038/oby.2007.198 17636083

[37] Laforest S, Michaud A, Paris G, et al. Comparative analysis of three human adipocyte size measurement methods and their relevance for cardiometabolic risk. Obesity [Internet] 2017 [cited 2025 Jan 31];25(1):122–131. doi: 10.1002/oby.21697 27883275

[38] Goffette V, Sabin N, Bugeon J, et al. Mature adipocytes inhibit differentiation of myogenic cells but stimulate proliferation of fibro-adipogenic precursors derived from trout muscle in vitro. Sci Rep [Internet]. 2024 [cited 2025 Jan 31];14(1):16422. Available from: https://www.nature.com/articles/s41598-024-67152-0 39013963 10.1038/s41598-024-67152-0 PMC11252293

[39] Maguire AS, Woodie LN, Judd RL, et al. Whole-slide image analysis outperforms micrograph acquisition for adipocyte size quantification. Adipocyte [Internet]. 2020 [cited 2025 Jan 31];9(1):567–575. doi: 10.1080/21623945.2020.1823139 32954932 PMC7714435

[40] Hagberg CE, Li Q, Kutschke M, et al. Flow cytometry of mouse and human adipocytes for the analysis of browning and cellular heterogeneity. Cell Rep [Internet] 2018 [cited 2025 Jan 31];24(10):2746–2756.e5. Available from: https://linkinghub.elsevier.com/retrieve/pii/S2211124718312609 30184507 10.1016/j.celrep.2018.08.006 PMC6137819

[41] Muller S, Ader I, Creff J, et al. Human adipose stromal-vascular fraction self-organizes to form vascularized adipose tissue in 3D cultures. Sci Rep [Internet]. 2019 [cited 2025 Jan 31];9(1):7250. Available from: https://www.nature.com/articles/s41598-019-43624-6 31076601 10.1038/s41598-019-43624-6 PMC6510792

[42] Chaudhuri O, Cooper-White J, Janmey PA, et al. Effects of extracellular matrix viscoelasticity on cellular behaviour. Nat [Internet]. 2020 [cited 2025 Jan 31];584(7822):535–546. Available from: https://www.nature.com/articles/s41586-020-2612-2 10.1038/s41586-020-2612-2 PMC7676152 32848221

[43] Awada M, Meynier A, Soulage CO, et al. N-3 PUFA added to high-fat diets affect differently adiposity and inflammation when carried by phospholipids or triacylglycerols in mice. Nutr Metab (lond) [Internet] 2013 [cited 2025 Aug 25];10(1):23. doi: 10.1186/1743-7075-10-23 23413782 PMC3585798

[44] Fryklund C, Borg M, Svensson T, et al. Impaired glucose transport in inguinal adipocytes after short-term high-sucrose feeding in mice. J Nutritional Biochem [internet]. 2020 [cited 2025 Aug 25];78:108338. Available from: https://linkinghub.elsevier.com/retrieve/pii/S095528631930693X 10.1016/j.jnutbio.2019.108338 32004930

[45] Lemonnier D. Effect of age, sex, and site on the cellularity of the adipose tissue in mice and rats rendered obese by a high-fat diet. J Clin Invest. 1972 [cited 2025 Aug 25];51(11):2907–2915. Available from: http://www.jci.org/articles/view/107115 5080416 10.1172/JCI107115 PMC292441

[46] Dufau J, Shen JX, Couchet M, et al. In vitro and ex vivo models of adipocytes. Am J Physiol-Cell Physiol [internet]. 2021 [cited 2025 Jan 31];320(5):C822–41. doi: 10.1152/ajpcell.00519.2020 33439778

[47] Jo J, Gavrilova O, Pack S, et al. Hypertrophy and/or hyperplasia: dynamics of adipose tissue growth. PLOS Comput Biol [Internet]. 2009 [cited 2025 Aug 25];5(3):e1000324. doi: 10.1371/journal.pcbi.1000324 19325873 PMC2653640

[48] Hemmeryckx B, Loeckx D, Dresselaers T, et al. Age-associated adaptations in murine adipose tissues. Endocr J [Internet] 2010 [cited 2025 Aug 25];57(10):925–930. Available from: http://www.jstage.jst.go.jp/article/endocrj/57/10/57_K10E-179/_article 20686275 10.1507/endocrj.k10e-179

[49] Morari J, Haddad-Tóvolli R, Silva Nogueira PA, et al. Body mass variability in age-matched outbred male Swiss mice is associated to differential control of food intake by ghrelin. Mol Cellular Endocrinol [Internet]. 2022 [cited 2025 Aug 25];550:111646. Available from: https://linkinghub.elsevier.com/retrieve/pii/S0303720722000946 35413387 10.1016/j.mce.2022.111646

[50] Lackey DE, Burk DH, Ali MR, et al. Contributions of adipose tissue architectural and tensile properties toward defining healthy and unhealthy obesity. Am J Physiol-Endocrinol Metab [internet]. 2014 [cited 2025 Jun 18];306(3):E233–46. doi: 10.1152/ajpendo.00476.2013 24302007 PMC3920015

[51] Albalat A, Gutiérrez J, Navarro I. Regulation of lipolysis in isolated adipocytes of rainbow trout (Oncorhynchus mykiss): the role of insulin and glucagon. Comp Biochem Physiol Part A. 2005 [cited 2025 Jan 31];142(3):347–354. Available from: https://linkinghub.elsevier.com/retrieve/pii/S1095643305002734 10.1016/j.cbpa.2005.08.006 16213761

[52] Pachitariu M, Stringer C. Cellpose 2.0: how to train your own model. Nat Methods [Internet]. 2022 [cited 2024 Nov 22];19(12):1634–1641. Available from: https://www.nature.com/articles/s41592-022-01663-4 36344832 10.1038/s41592-022-01663-4 PMC9718665

