# Supplementary material for: 3D imaging and quantitative analysis of adipocytes in situ and ex situ
Source: Adipocyte. 2025 Sep 21;14(1):2558573. doi: 10.1080/21623945.2025.2558573 (PMC12456214; doi:10.1080/21623945.2025.2558573)
Supplement: Suppdata1.docx [file KADI_A_2558573_SM3749.docx]

| **structure** | **product** | **producer** | **reference** | **excitation** | **in situ** | **ex situ** |
| --- | --- | --- | --- | --- | --- | --- |
|  |  |  |  |  |  |  |
|  | Bodipy | Fisher scientific | 11540326 | 488 nm | 1/1000 | 1/800 |
| lipids | Nile red | Thermo Fisher | N1142 | 552 nm | 1/500 | 1/500 |
|  | Lipidtox | Thermo Fisher | H34476 | 594 nm | 1/1000 | 1/1000 |
|  |  |  |  |  |  |  |
|  | WGA green | Thermo Fisher | W11261 | 495 nm | 1/500 | Not Done |
| membrane | Membrite | Biotium | 30093 | 515 nm | 1/1000 | 1/1000 |
|  | Cell Mask deep red | Thermo Fisher | C10046 | 649 nm | 1/1000 | 1/800 |
|  |  |  |  |  |  |  |
| ECM | 5-DTAF | Invitrogen | 11550216 | 488 nm | 200µg/ml | NO ECM |
|  |  |  |  |  |  |  |
|  | Hoechst | Thermo Fisher | 33342 | UV | 1/1000 | 1/500 |
| DNA/nucleus | DAPI | Sigma | D8417 | UV | 1/1000 | 1/500 |
