# Supplementary figures and images for: 3D imaging and quantitative analysis of adipocytes in situ and ex situ
Source: Adipocyte. 2025 Sep 21;14(1):2558573. doi: 10.1080/21623945.2025.2558573 (PMC12456214; doi:10.1080/21623945.2025.2558573)

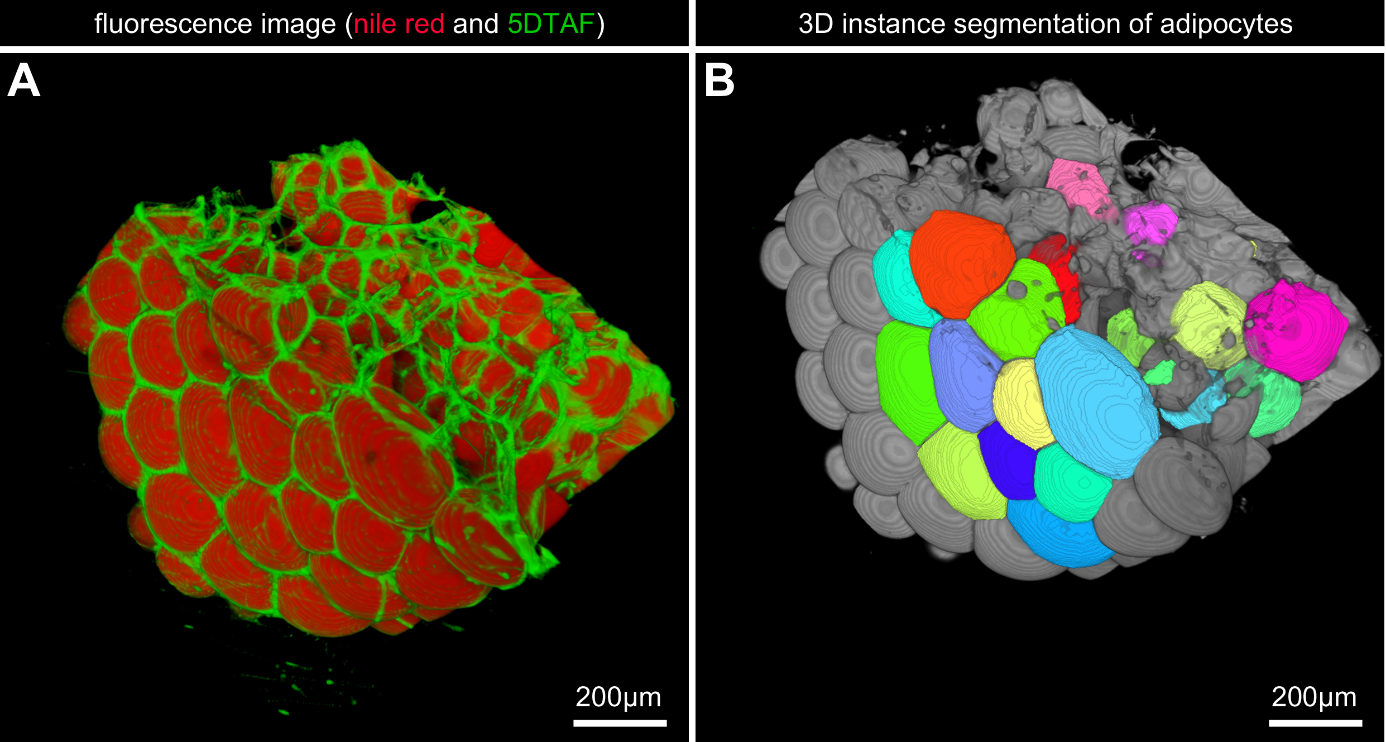

Supplement: Suppdata4.docx [file KADI_A_2558573_SM3748.docx]

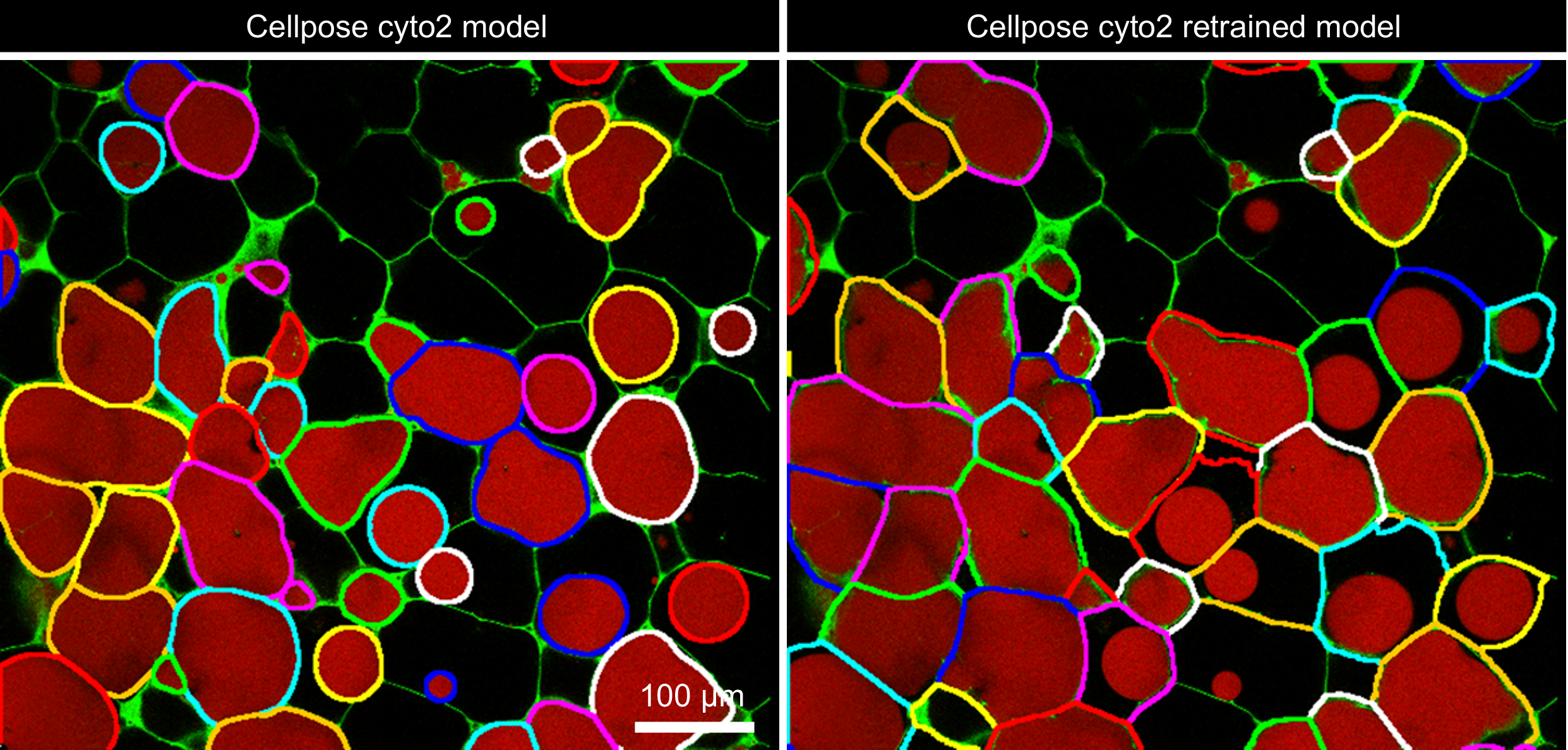

Supplement: suppdata3.tif [file KADI_A_2558573_SM3746.tif]

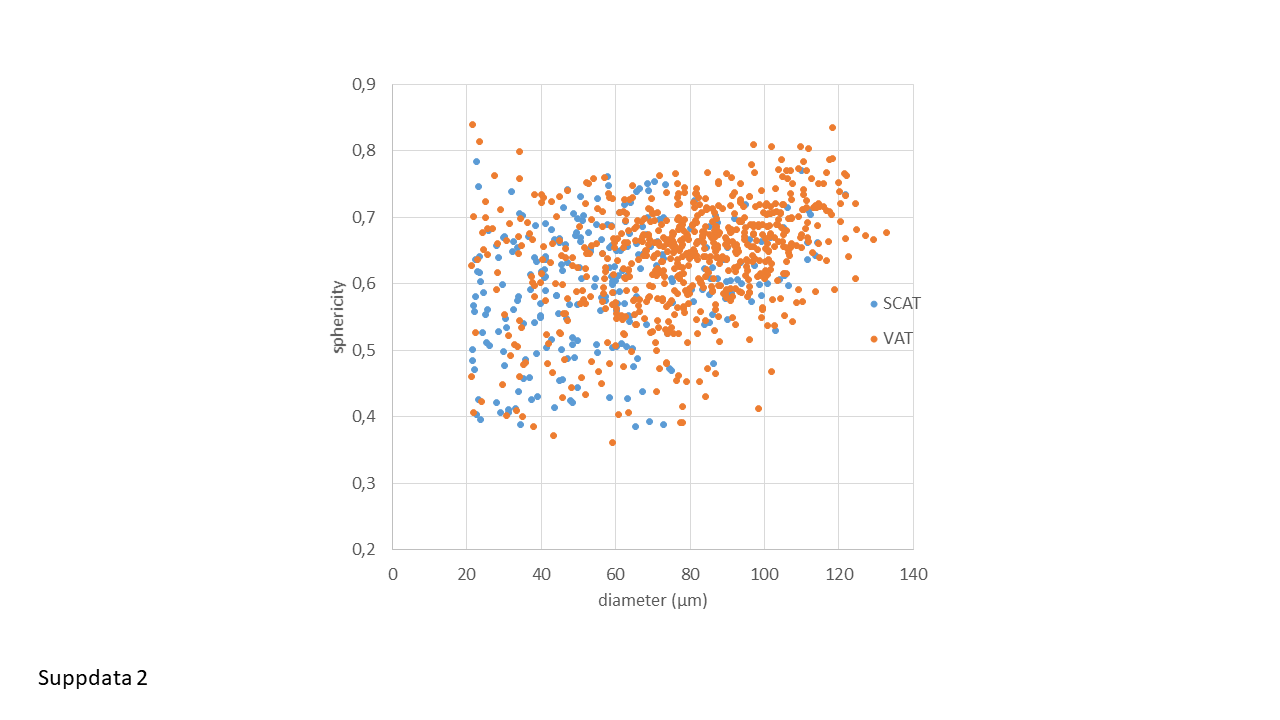

Supplement: suppdata2.tif [file KADI_A_2558573_SM3745.tif]
