# Supplementary material for: 3D imaging and quantitative analysis of adipocytes in situ and ex situ
Source: Adipocyte. 2025 Sep 21;14(1):2558573. doi: 10.1080/21623945.2025.2558573 (PMC12456214; doi:10.1080/21623945.2025.2558573)
Supplement: Suppdata5.docx [file KADI_A_2558573_SM3747.docx]

2

|  | | | | | | | | | | | | |
| --- | --- | --- | --- | --- | --- | --- | --- | --- | --- | --- | --- | --- |
|  |  | | | | **Tissue weight once dissected (g)** | | | | **ex situ adipocytes (x106/g of tissue)** | | | |
|  | | | | | | | | | | | | |
| **Experiments** | **Fish weight (g), sex** | | | | VAT | | SCAT | | VAT | | SCAT | |
|  |  |  |  |  |  | | | | | | | |
| M12 | 559,00 | M | 919,00 | F | 10,57 | 11,54 | 1,67 | 2,50 | 2,30 | 2,50 | 5,80 | 8,20 |
| M13 | 874,00 | M | 1407,00 | F | 10,24 | 14,81 | 2,19 | 3,75 | 0,64 | 0,50 | 1,60 | 24,00 |
| M14 | 663,00 | F | 1342,00 | M | 6,34 | 22,39 | 3,36 | 4,60 | 0,93 | 0,82 | 6,70 | 6,60 |
| M15 | 754,00 | M | 860,00 | F | 7,50 | 10,19 | 1,45 | 1,94 | 4,00 | 1,40 | 0,89 | 8,70 |
| M16 | 833,00 | M | 1250,00 | M | 6,75 | 17,27 | 1,86 | 2,99 | 0,02 | 0,99 | 5,60 | 4,30 |
| M17 | 810,00 | F | 1089,00 | M | 5,27 | 17,70 | 2,04 | 3,43 | 0,09 | 4,90 | 0,61 | 7,60 |
| M18 | 815,00 | F | 1309,00 | M | 13,71 | 17,44 | 3,46 | 6,17 | 3,30 | 1,00 | 8,60 | 9,30 |
| M19 | 665,00 | F | 1052,00 | F | 6,59 | 15,82 | 1,50 | 4,04 | 1,40 | 2,20 | 0,87 | 14,00 |
|  | | | | | | | | | | | | |
| **Fish batch (kg)** | 0,75 | | 1,10 | | 0,75 | 1,10 | 0,75 | 1,10 | 0,75 | 1,10 | 0,75 | 1,10 |
